# Establishment of a prognostic model for gastric cancer patients who underwent radical gastrectomy using machine learning: a two-center study

**DOI:** 10.3389/fonc.2023.1282042

**Published:** 2024-04-11

**Authors:** Tong Lu, Miao Lu, Haonan Liu, Daqing Song, Zhengzheng Wang, Yahui Guo, Yu Fang, Qi Chen, Tao Li

**Affiliations:** ^1^ Department of Emergency Medicine, Jining No.1 People’s Hospital, Jining, China; ^2^ Wuxi Mental Health Center, Wuxi, China; ^3^ Department of Oncology, Affiliated Hospital of Xuzhou Medical University, Xuzhou, China; ^4^ Department of Gastroenterology, The Second Affiliated Hospital of Xuzhou Medical University, Xuzhou, China; ^5^ Department of Gastroenterology, Xuzhou First People’s Hospital, Xuzhou, China; ^6^ Jiangsu Normal University, Xuzhou, China; ^7^ Department of Gastroenterology, Jining First People’s Hospital, Jining, China

**Keywords:** machine learning, gastric cancer, prognosis, clinical model, nomogram model

## Abstract

**Objective:**

Gastric cancer is a prevalent gastrointestinal malignancy worldwide. In this study, a prognostic model was developed for gastric cancer patients who underwent radical gastrectomy using machine learning, employing advanced computational techniques to investigate postoperative mortality risk factors in such patients.

**Methods:**

Data of 295 patients with gastric cancer who underwent radical gastrectomy at the Department of General Surgery of Affiliated Hospital of Xuzhou Medical University (Xuzhou, China) between March 2016 and November 2019 were retrospectively analyzed as the training group. Additionally, 109 patients who underwent radical gastrectomy at the Department of General Surgery Affiliated to Jining First People’s Hospital (Jining, China) were included for external validation. Four machine learning models, including logistic regression (LR), decision tree (DT), random forest (RF), and gradient boosting machine (GBM), were utilized. Model performance was assessed by comparing the area under the curve (AUC) for each model. An LR-based nomogram model was constructed to assess patients’ clinical prognosis.

**Results:**

Lasso regression identified eight associated factors: age, sex, maximum tumor diameter, nerve or vascular invasion, TNM stage, gastrectomy type, lymphocyte count, and carcinoembryonic antigen (CEA) level. The performance of these models was evaluated using the AUC. In the training group, the AUC values were 0.795, 0.759, 0.873, and 0.853 for LR, DT, RF, and GBM, respectively. In the validation group, the AUC values were 0.734, 0.708, 0.746, and 0.707 for LR, DT, RF, and GBM, respectively. The nomogram model, constructed based on LR, demonstrated excellent clinical prognostic evaluation capabilities.

**Conclusion:**

Machine learning algorithms are robust performance assessment tools for evaluating the prognosis of gastric cancer patients who have undergone radical gastrectomy. The LR-based nomogram model can aid clinicians in making more reliable clinical decisions.

## Introduction

Gastric cancer (GC) is believed to be the fifth most common cancer and the third most common cause of death worldwide.Notably, China and Japan are at the forefront, collectively accounting for 75% of Asian cases (1, 2). Despite being one of the most common treatment modalities for gastric cancer, surgical intervention alone has failed to elevate the overall 5-year survival rate beyond 50%. Thus, the quest for precise clinical assessments holds paramount clinical importance for the diagnosis and management of affected patients (3). One widely embraced approach in clinical research involves amassing clinical data to construct prognostic models. Within this domain, gastric cancer model studies have proliferated, offering the promise of better-informed clinical decision-making (4, 5). In addition to clinicopathological data, these models incorporate hematologic inflammatory markers and the widely utilized carcinoembryonic antigen (CEA). The association between inflammation and its impact on the occurrence, progression, metastasis, and prognosis of cancer patients, as revealed by blood-based metrics, has become a burgeoning area of research interest (6, 7). The principle underlying the utilization of CEA as a serum tumor marker is well-established in clinical practice. This marker finds extensive utility in the early screening of various tumors. Furthermore, its early elevation is recognized as an independent risk factor associated with the poorer prognosis of gastric cancer (8).

Machine learning stands as a precision algorithm within the context of artificial intelligence, uniquely poised to decipher vast and intricate medical datasets. Its capacity to construct clinical prediction models makes it an invaluable tool in the realm of healthcare, offering crucial assistance in diagnosis and prognostication (9). The development of clinical predictive models typically involves the processing and optimization of large datasets within a training set. Subsequently, these models undergo rigorous testing using external validation set data, a pivotal step in establishing their external validity and, by extension, their applicability to diverse patient populations (10, 11). Cancer, marked by its complexity and heterogeneity, emerges as a particularly promising frontier for machine learning applications in medical research. The significance of clinical data available empowers early cancer detection, facilitates ongoing monitoring of disease progression, and supports the optimization of treatment strategies (9, 12).

## Patients and methods

### Patients’ enrollment

**Figure 1:**
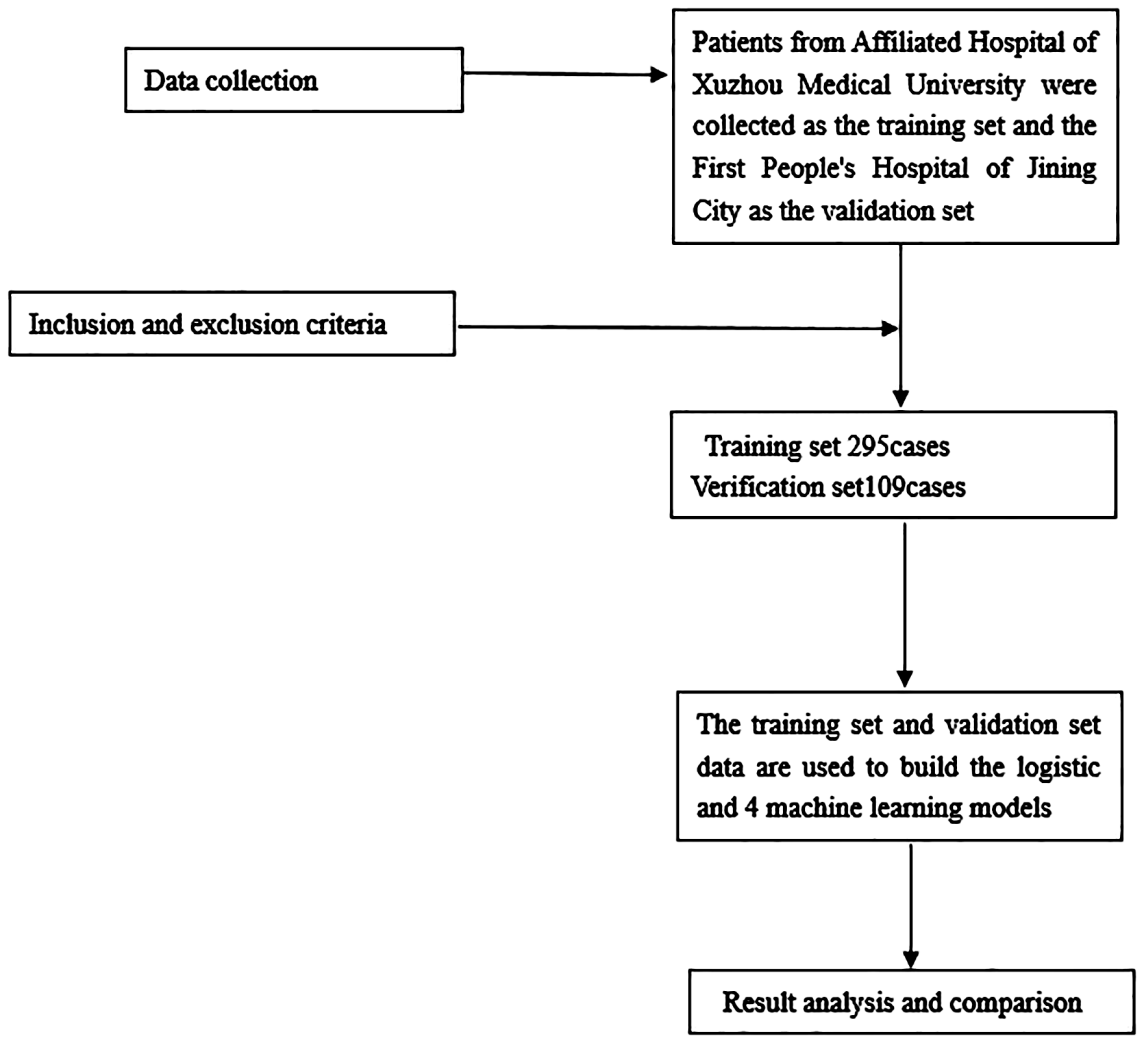
Flowchart of patients’ selection.

This retrospective analysis involved a total of 295 gastric cancer patients who underwent radical gastrectomy at the Department of General Surgery, Affiliated Hospital of Xuzhou Medical University (Xuzhou, China), between March 2016 and November 2019. These patients constituted the training group. Additionally, 109 gastric cancer patients who underwent radical gastrectomy at the Department of General Surgery of Jining First People’s Hospital (Jining, China) were included as the verification group. The inclusion criteria were as follows: (1): patients newly diagnosed with gastric cancer, for whom comprehensive medical records were available; (2) cases where primary radical resection of gastric cancer was conducted at the respective hospitals, with subsequent confirmation of gastric adenocarcinoma; (3) absence of any prior anti-tumor therapies, including radiotherapy or chemotherapy, before surgical intervention. The exclusion criteria were as follows: (1) patients with concurrent malignancies; (2) patients presenting preoperative complications of other infectious diseases, blood system disorders, autoimmune conditions, or any other medical conditions that could potentially influence inflammatory markers; (3) cases who had recently received or were currently undergoing anti-inflammatory or immunosuppressive treatments; (4) patients subjected to preoperative blood transfusion therapy; (5) patients with severe liver or kidney dysfunction; (6) cases featuring incomplete clinical data or visitor information. Further details are illustrated in **Figure 1**.

### Outcome measures

The primary outcome event for this study was the survival status of patients at the three-year post-radical gastrectomy. Follow-up procedures involved telephonic or outpatient monitoring. The survival rate was determined from the date of admission to either the date of decease or the specified deadline for follow-up.

### Research purpose

This study concentrated on evaluating the three-year survival outcomes of patients who underwent radical gastrectomy. A total of 404 gastric cancer patients from two medical centers were included in the study. A machine learning algorithm was employed to develop a clinical prediction model aimed at identifying the prognostic risk factors for postoperative patients. The creation of a visual nomogram model, based on these risk factors, can aid healthcare professionals in conducting risk assessments.

### Risk factors

Concerning the study subjects, clinical data were collected, including patient’s name, age, gender, and clinicopathological information. This included data on blood parameters, tumor location, maximum tumor size, TNM stage, lymph node involvement, nerve vessel invasion, method of gastrectomy, tumor differentiation grade, along with specific blood markers including neutrophil count, monocyte count, lymphocyte count, and CEA level. Peripheral venous blood samples were obtained from fasting cases on the next morning. The collected indices were then incorporated into the Lasso regression model. The Lasso model employs a technique that can shrink the coefficients of unimportant variables to 0, promoting feature selection. Following the establishment of inclusion and exclusion criteria, the relevant data were fed into the Lasso model, enabling the complete elimination of the weight associated with the least important variables. This process allows for data screening and complexity adjustment while fitting the generalized linear model. Consequently, the Lasso model ensures the accuracy of variables in the subsequent development of the machine learning model.

### Statistical analysis

Continuous variables were presented as mean ± standard deviation, and categorical variables were expressed as ratio. To create the machine learning and nomogram models, the process was initiated by applying a Lasso regression model to identify the key risk factors linked to the 3-year survival status of patients, as depicted in **Figure 1**. Subsequently, these relevant risk factors were integrated into machine learning algorithms, leading to the development of logistic regression (LR), decision tree (DT), random forest (RF), and gradient boosting machine (GBM) models. Model performance was assessed by comparing the area under the curve (AUC) of each model. Ultimately, a LR model was selected to construct a nomogram, enhancing the interpretability and visibility of the results.

### Feature selection and machine learning performance evaluation

**Figure 2:**
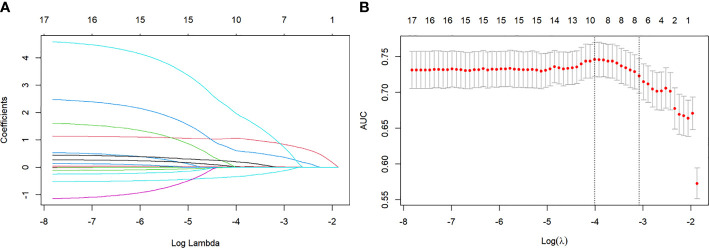
**(A)** Lasso regression coefficient path diagram. Lasso regression variables were used for dimensionality reduction to further screen the relevant variables. **(B)** Lasso regression cross validation. Using ten-fold cross-validation, the λ value with the smallest cross-validation error is used as the optimal solution of the model.

**Figure 3:**
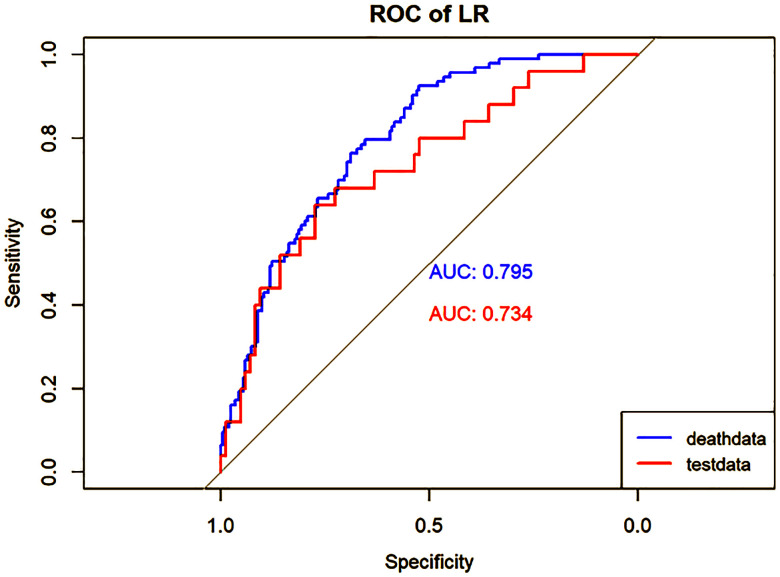
Performance of the LR model. The AUC, Sen and Spe of the training and internal validation sets were exhibited in figure, respectively. ROC, receiver operating characteristic; AUC, area under the curve; Sen, sensitivity; Spe, specificity. Blue line: Training set. Red line: Validation set.

**Figure 4:**
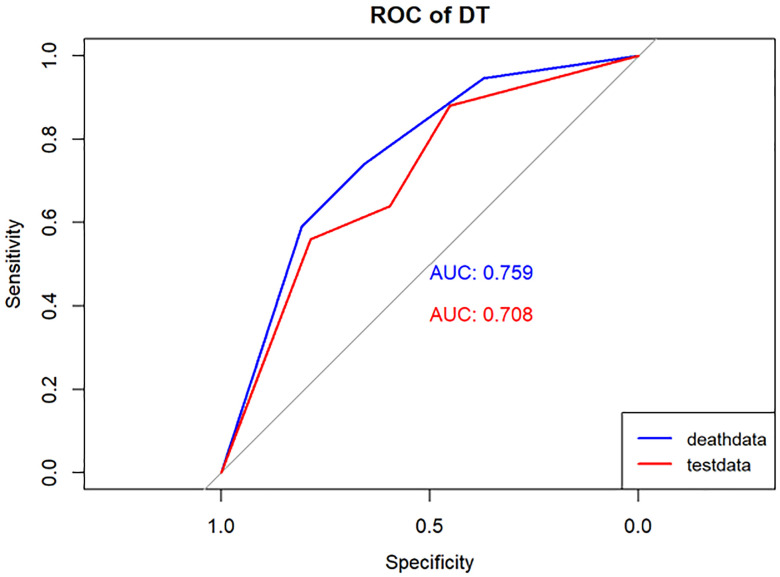
Performance of the DT model. The AUC, Sen and Spe of the training and internal validation sets were exhibited in figure, respectively. ROC, receiver operating characteristic; AUC, area under the curve; Sen, sensitivity; Spe, specificity. Blue line: Training set. Red line: Validation set.

**Figure 5:**
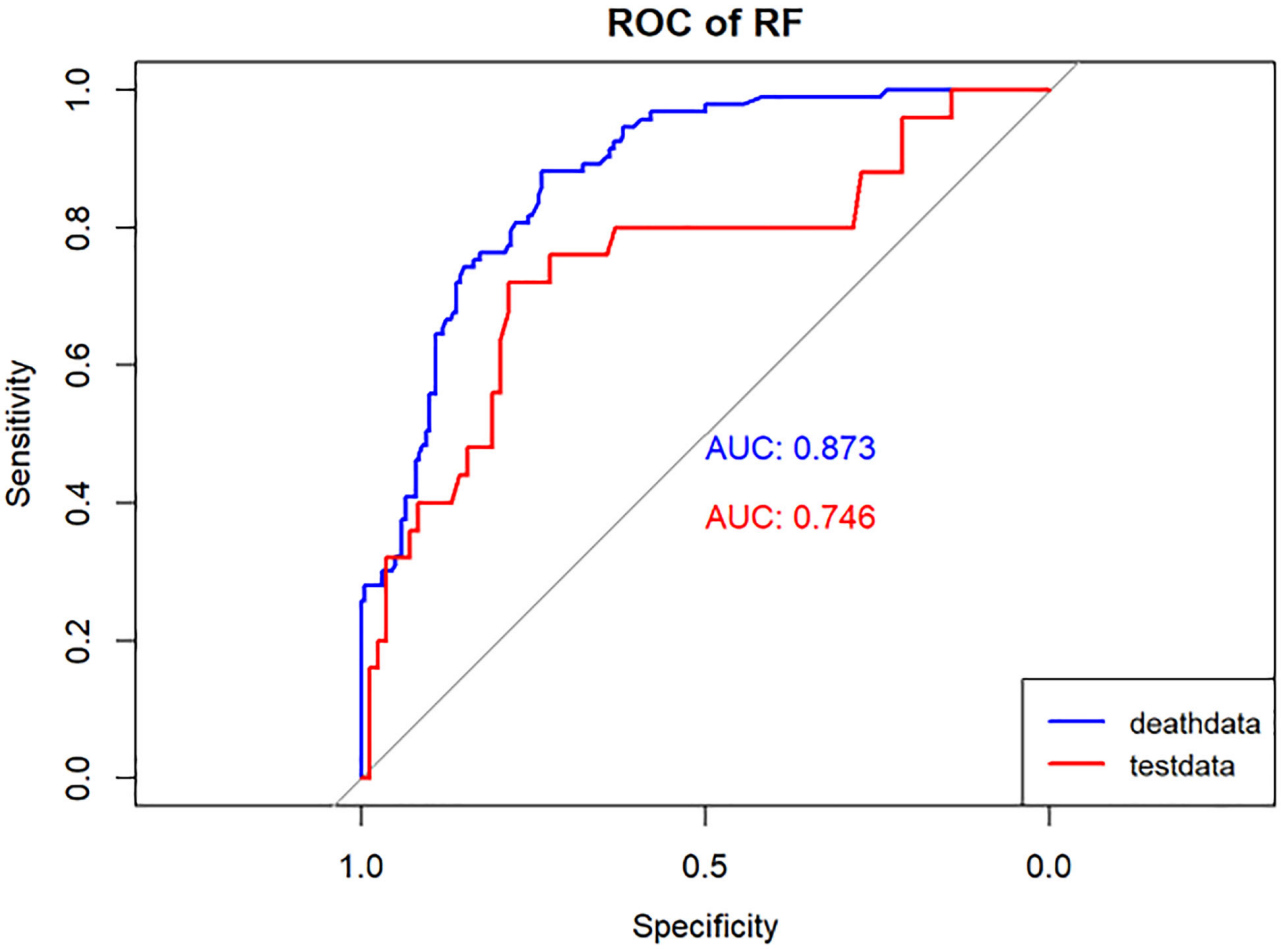
Performance of the RF model. The AUC, Sen and Spe of the training and internal validation sets were exhibited in figure, respectively. ROC, receiver operating characteristic; AUC, area under the curve; Sen, sensitivity; Spe, specificity. Blue line: Training set. Red line: Validation set.

**Figure 6:**
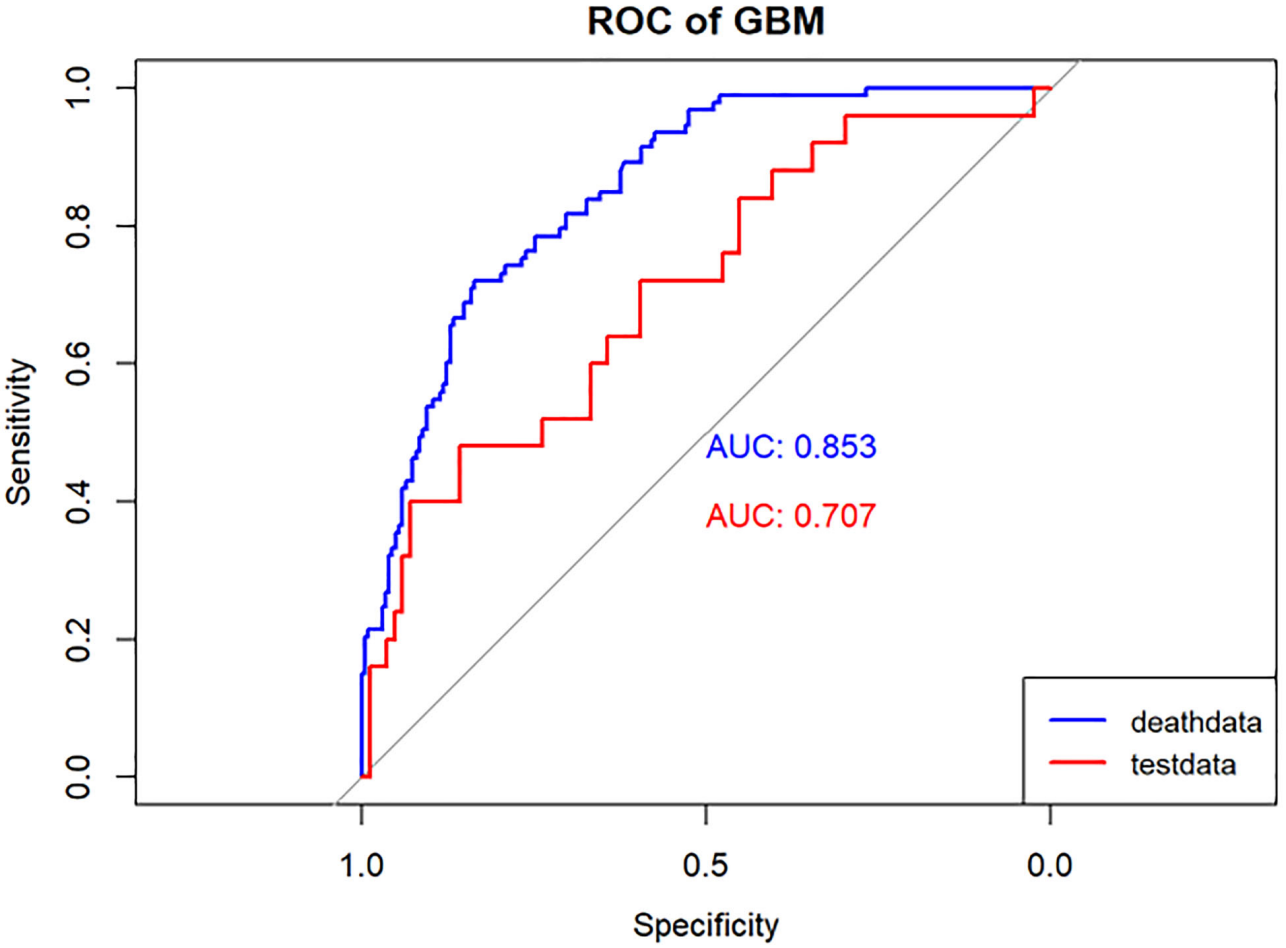
Performance of the GBM model. The AUC, Sen and Spe of the training and internal validation sets were exhibited in figure, respectively. ROC, receiver operating characteristic; AUC, area under the curve; Sen, sensitivity; Spe, specificity. Blue line: Training set. Red line: Validation set.

To reduce model complexity and eliminate redundant or irrelevant data in the training group, we applied the Lasso regression model to screen the variables, as illustrated in **Figures 2A**, **B**. Besides, 4 machine learning models (LR, DT, RF, and GBM), as illustrated in **Figures 3**–**6** were used in this study. LR is a classification algorithm that seeks to establish a relationship between a feature and the probability of a specific outcome. It possesses the advantage of not presupposing the data distribution and presents results in a probabilistic format, making it appropriate for numerous probability-assisted decision-making tasks. Nonetheless, LR proves ineffective for handling nonlinear data and exhibits heightened sensitivity to imbalances in multicollinearity datasets (13, 14). DT is primarily used for classification tasks, and decision trees start from a root node to identify the initial decision point in a dataset and contain features that best divide the dataset into distinct classes. DT is well-suited for handling irrelevant features, offering a model that is easy to understand and explain. They can be visualized and analyzed, facilitating a clear interpretation of the underlying rules. Additionally, DT is effective in dealing with missing data (15). RF, as an extension of the DT method, combines multiple DTs, with the majority vote among the trees determining the final class prediction of the model. RF incurs a substantial training cost, and the decision-making process of the model is susceptible to the specific division of feature values (16, 17). GBM is a boosting technique utilized as a numerical optimization algorithm for minimizing loss functions and constructing additive models. It proves effective for small-scale datasets, excelling in the processing of multi-classification tasks and accommodating incremental training. Additionally, GBM demonstrates good inclusiveness for handling missing data. However, its performance diminishes when dealing with high-dimensional feature spaces. The effectiveness of GBM in classification tasks is also reliant on the division of feature attributes, making it more sensitive to the expression form of input data (18, 19).

Model performance was evaluated using various metrics, including accuracy, recall, and the area under the ROC curve, a primary indicator for binary classification performance, ranging from 0 to 1, with higher values signifying superior performance. Additionally, for models with two outcomes, we reported the area under the accuracy-recall curve, which illustrates the trade-off between true accuracy and positive predicted values, as well as the F1 score, defined as the harmonic mean of precision and recall. The models underwent 10-fold cross-validation on the training set and were subsequently tested on the test set, as shown in **Tables 1** and **2**.

**Table 1:** The model performance in the training dataset.

model	AUC	Accuracy	Sensitivity (Recall Rates)	Specificity
LR	0.795	0.712	0.763	0.688
DT	0.759	0.739	0.591	0.807
RF	0.873373	0.783	0.882	0.738
GBM	0.863	0.800	0.720	0.837

**Table 2:** The model performance in the validation dataset.

model	AUC	Accuracy	Sensitivity (Recall Rates)	Specificity
LR	0.734	0.697	0.680	0.702
DT	0.708	0.733	0.560	0.786
RF	0.746	0.670	0.760	0.643
GBM	0.707	0.716	0.480	0.786

### Nomogram

**Figure 7:**
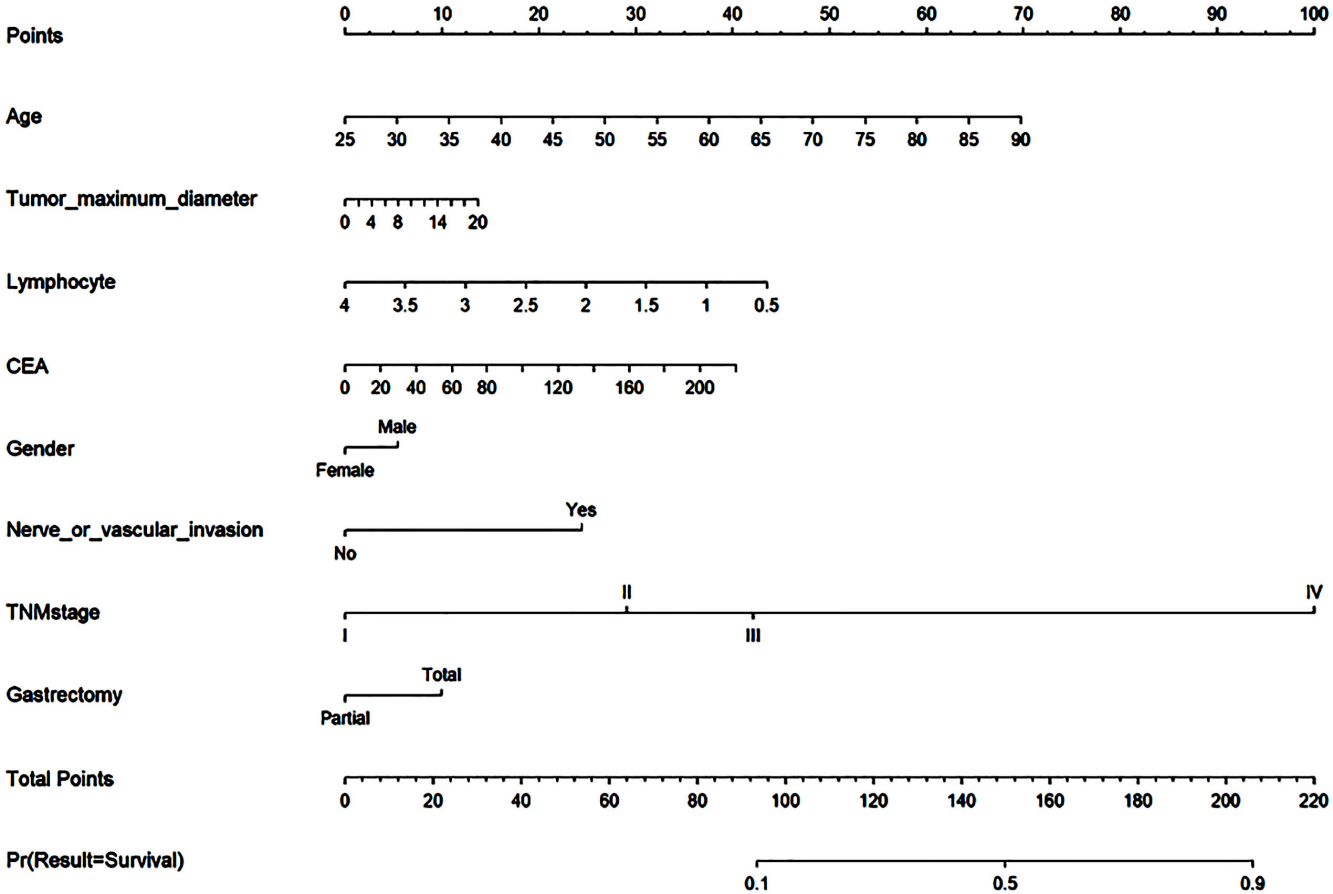
Nomogram. Lines 2 through 9 in the nomogram represent the risk scores associated with individual patients. The cumulative score serves as an indicator for assessing patients’ prognoses, with higher scores signifying an increased risk level and a poorer prognosis.

LR was employed to construct a nomogram model for predicting the risk of mortality following radical gastrectomy, utilizing eight variables incorporated into the model. Lines 2 through 9 in the nomogram represent the risk scores associated with individual patients, as shown in **Figure 7**. The cumulative score serves as an indicator for assessing patients’ prognoses, with higher scores signifying an increased risk level and a poorer prognosis.

## Results

### Patients’ baseline characteristics

Patients’ baseline characteristics are presented in **Table 3**. The training group consisted of 295 patients, among whom 93 (73 males and 20 females) passed away within 3 years. The validation group comprised 109 patients, with 25 fatalities (14 males and 11 females). In the training group, variables, such as age, maximum tumor diameter, TNM stage, lymph node metastasis, nerve or vascular invasion, type of gastrectomy, lymphocyte count, and CEA level exhibited statistically significant differences between patients who survived and those who succumbed. Conversely, there were no statistically significant differences in gender, tumor differentiation, tumor site, neutrophil count, and monocyte count. In the validation group, significant differences were found in maximum tumor diameter, TNM stage, lymph node metastasis, and nerve or vascular invasion, while other variables did not exhibit significant differences.

**Table 3:** Patients’ baseline characteristics.

	Training set				Validation set			
	Overall	Survival	Death	P	Overall	Survival	Death	P
	*N=295*	*N=202*	*N=93*		*N=109*	*N=84*	*N=25*	
Age	59.70±11.70	58.13±11.89	63.12±10.55	<0.001	60.88±10.29	60.96±10.41	60.60±10.07	0.876
Gender:				0.134				0.114
Male	213 (72.20%)	140 (69.31%)	73 (78.49%)		77 (70.64%)	63 (75.00%)	14 (56.00%)	
Female	82 (27.80%)	62 (30.69%)	20 (21.51%)		32 (29.36%)	21 (25.00%)	11 (44.00%)	
Tumor maximum diameter (cm)	4.93±3.09	4.40±2.91	6.06±3.19	<0.001	4.12±2.55	3.61±2.17	5.84±3.01	0.002
TMN stage:				<0.001				<0.001
I	80 (27.12%)	75 (37.13%)	5 (5.38%)		41 (37.61%)	38 (45.24%)	3 (12.00%)	
II	63 (21.36%)	45 (22.28%)	18 (19.35%)		23 (21.10%)	21 (25.00%)	2 (8.00%)	
III	146 (49.49%)	81 (40.10%)	65 (69.89%)		41 (37.61%)	24 (28.57%)	17 (68.00%)	
IV	6 (2.03%)	1 (0.50%)	5 (5.38%)		4 (3.67%)	1 (1.19%)	3 (12.00%)	
Lymph node metastasis:				<0.001				0.020
No	105 (35.59%)	87 (43.07%)	18 (19.35%)		46 (42.20%)	41 (48.81%)	5 (20.00%)	
Yes	190 (64.41%)	115 (56.93%)	75 (80.65%)		63 (57.80%)	43 (51.19%)	20 (80.00%)	
Nerve or vascular invasion:				<0.001				0.026
No	111 (37.63%)	98 (48.51%)	13 (13.98%)		45 (41.28%)	40 (47.62%)	5 (20.00%)	
Yes	184 (62.37%)	104 (51.49%)	80 (86.02%)		64 (58.72%)	44 (52.38%)	20 (80.00%)	
Degree of differentiation:				0.355				0.106
Low	140 (47.46%)	92 (45.54%)	48 (51.61%)		79 (72.48%)	57 (67.86%)	22 (88.00%)	
Moderate	142 (48.14%)	99 (49.01%)	43 (46.24%)		20 (18.35%)	17 (20.24%)	3 (12.00%)	
High	13 (4.41%)	11 (5.45%)	2 (2.15%)		10 (9.17%)	10 (11.90%)	0 (0.00%)	
Tumor site:				0.650				0.052
Cardia	103 (34.92%)	67 (33.17%)	36 (38.71%)		19 (17.43%)	12 (14.29%)	7 (28.00%)	
Gastric antrum	145 (49.15%)	102 (50.50%)	43 (46.24%)		53 (48.62%)	39 (46.43%)	14 (56.00%)	
Gastric body	47 (15.93%)	33 (16.34%)	14 (15.05%)		37 (33.94%)	33 (39.29%)	4 (16.00%)	
Gastrectomy:				0.008				0.182
Partial	209 (71.09%)	153 (76.12%)	56 (60.22%)		79 (72.48%)	64 (76.19%)	15 (60.00%)	
Total	85 (28.91%)	48 (23.88%)	37 (39.78%)		30 (27.52%)	20 (23.81%)	10 (40.00%)	
Neutrophil count	3.80±1.50	3.79±1.54	3.82±1.43	0.882	3.19±1.07	3.22±1.08	3.07±1.03	0.527
Lymphocyte count	1.76±0.54	1.82±0.55	1.63±0.48	0.004	1.68±0.77	1.71±0.82	1.57±0.55	0.324
Monocyte count	0.39±0.15	0.38±0.15	0.40±0.17	0.445	0.42±0.20	0.43±0.22	0.39±0.11	0.211
CEA	8.74±23.57	6.46±17.15	13.70±33.12	0.049	5.98±10.93	4.50±5.12	10.96±20.34	0.128

## Discussion

Machine learning employs computer algorithms to identify intricate relationships or patterns within extensive datasets. It accomplishes this by performing numerous operations using pre-existing algorithms to recognize and analyze data. Through iterative adjustments to these algorithms, machine learning strives to achieve optimal performance, resulting in the creation of models that establish connections between multiple variables and target variables (20). In essence, supervised machine learning is tasked with identifying associations between input and output data, enabling the prediction of outcomes based on patients’ data (21). Machine learning represents a fundamental shift in healthcare, where computers glean insights from patient data without the need for explicit programming of specific tasks. This approach possesses the advantages of enhanced capacity, objectivity, and repeatability when handling large datasets, thereby ensuring data reliability (22, 23). It has the potential to enhance the quality of early diagnosis, disease progression monitoring, and the ability to predict patient-specific outcomes in orthopedics, such as prognosis, risk of complications, and implant longevity (24). These advantages promote the sharing of decision-making information between healthcare professionals and patients, facilitating effective planning and rational utilization of healthcare services (25, 26). In addition, the model can be periodically retrained to improve prediction accuracy over time (27).

In the present study, Lasso regression was employed to identify 8 risk factors associated with postoperative mortality in gastric cancer patients. Additionally, we established four machine learning models to assess patient prognosis and created nomograms to evaluate prognosis based on LR. Lasso regression effectively filtered out non-statistically significant variables during the variable screening process, thereby reducing data redundancy and enhancing the model’s accuracy and reliability by using fewer variables. This approach to developing clinical models has found applications in various medical domains (28, 29). The models’ performance was assessed using the ROC curve, with metrics, such as AUC values, sensitivity, specificity, and accuracy. **Table 1** illustrates that all four models exhibit commendable accuracy, indicating the robust diagnostic capability of the machine learning models for predicting postoperative prognosis in gastric cancer patients. **Table 2** further validates these findings in the verification group, demonstrating the models’ strong external applicability. Collectively, these results underscore the effectiveness of machine learning models in accurately reflecting postoperative outcomes in gastric cancer surgery (30, 31).

The postoperative prognosis histogram provides an intuitive representation of prognostic risk in gastric cancer patients. **Figure 7** illustrates specific scores assigned to variables including age, gender, lymphocyte count, maximum tumor diameter, CEA level, nerve or vascular invasion, TNM stage, and gastrectomy method. In the previous study, Hu used traditional methods to establish clinical models to prove positive LNs, tumor size, adjacent organs invasion, vascular invasion, CA125, the depth of invasion, and HER2 status is the reason that affects radical gastrectomy (32). In the model established by our machine learning algorithm, age and gender are also proved to be the factors that affect the prognosis of radical gastrectomy, which exactly proves that the machine learning algorithm has more powerful computing power.

A nomogram serves as a valuable tool for stratifying the risk of patients, enabling clinicians to assess their conditions effectively. This model assigns scores to various characteristic variables, allowing clinicians to evaluate a patient’s status based on these characteristics. Higher scores on the nomogram indicate an increased susceptibility to risk and a less favorable prognosis. Consequently, patients with distinct scores can benefit from tailored treatment strategies, ensuring a more personalized approach to their healthcare. For instance, determining whether to administer chemotherapy to postoperative gastric cancer patients is typically based on clinical recommendations for patients in stage 1b to stage 3. However, the decision regarding when to initiate chemotherapy for stage 1b to stage 3 patients can be informed by the risk score derived from the histogram. Among patients at the same stage, those with higher scores may be advised to pursue additional treatments. This approach effectively stratifies patients based on their individual conditions, facilitating personalized diagnosis and treatment.

The model identified 8 risk factors for postoperative death in gastric cancer patients using Lasso regression. In addition, 4 machine learning models were developed to assess patient prognosis and nomograms were established based on LR to predict patients’ outcomes. Lasso regression effectively filtered out irrelevant factors, reducing data redundancy, and enhancing model accuracy and reliability with fewer variables. This approach has been applied in various medical fields.

## Limitation

There are certain limitations in this study. The retrospective nature of the study may introduce subjective and selective biases,The reliability and validity of the data are limited, and we cannot completely eliminate the possibility of selection bias. Moreover, despite being a two-center study, the sample size remains relatively limited. Further validation with large-scale research is essential to confirm the model’s external applicability.

## Conclusions

In conclusion, age, gender, lymphocyte count, maximum tumor diameter, CEA level, nerve or vascular invasion, TNM stage, and gastrectomy method could serve as risk factors influencing the postoperative survival of gastric cancer patients. The machine learning model, established through Lasso regression, demonstrated promising performance and reliability. The nomogram model, which is based on the LR model, provides a practical tool for individualized diagnosis and treatment in clinical settings.

## Data availability statement

The raw data supporting the conclusions of this article will be made available by the authors, without undue reservation.

## Ethics statement

The studies involving humans were approved by Affiliated Hospital of Xuzhou Medical University. The studies were conducted in accordance with the local legislation and institutional requirements. The ethics committee/institutional review board waived the requirement of written informed consent for participation from the participants or the participants’ legal guardians/next of kin because this study protocol was approved by the Ethics Committee.

## Author contributions

TLu: Writing – original draft. ML: Writing – review & editing. HL: Methodology, Writing – review & editing. DS: Writing – review & editing. ZW: Data curation, Writing – review & editing. YG: Data curation, Writing – review & editing. YF: Data curation, Writing – review & editing. QC: Supervision, Writing – review & editing. TLi: Investigation, Writing – review & editing.
